# Association between Serum 25-Hydroxy-Vitamin D and Aggressive Prostate Cancer in African American Men

**DOI:** 10.3390/nu9010012

**Published:** 2016-12-28

**Authors:** Shakira M. Nelson, Ken Batai, Chiledum Ahaghotu, Tanya Agurs-Collins, Rick A. Kittles

**Affiliations:** 1 Cancer Prevention Fellow, Division of Cancer Prevention, National Cancer Institute, 9609 Medical Center Drive, Room 6E402, Bethesda, MD 20892, USA; 2 Metabolic Epidemiology Branch, Division of Cancer Epidemiology and Genetics, National Cancer Institute, 9609 Medical Center Drive, Room 6E402, Bethesda, MD 20892, USA; 3 Division of Urology, Department of Surgery, The University of Arizona, Tucson, AZ 85721, USA; kbatai@email.arizona.edu (K.B.); rkittles@surgery.arizona.edu (R.A.K.); 4 Chief Medical Officer, Carney Hospital-Steward Health Systems, Dorchester, MA 02124, USA; chiledum.ahaghotu@steward.org; 5 Health Behavior Research Branch, Division of Cancer Control and Population Studies, National Cancer Institute, 9609 Medical Center Drive, Bethesda, MD 20892, USA; collinsta@mail.nih.gov

**Keywords:** serum 25-hydroxyvitamin D, *rs11568820*, calcium, African American men, aggressive prostate cancer, vitamin D receptor small nucleotide polymorphisms

## Abstract

African American men have higher incidence rates of aggressive prostate cancer, where high levels of calcium and serum vitamin D deficient levels play a role in the racial differences in incidence. In this study, we examined associations of serum vitamin D with aggressive prostate cancer to improve our understanding of higher susceptibility of aggressive disease in this racial cohort. From Howard University Hospital, 155 African American men with clinically-identified prostate cancer were identified; 46 aggressive cases, and 58 non-aggressive cases. Serum vitamin D was assessed from fasting blood samples, and total calcium intake was assessed using the Block Food Frequency Questionnaire. Vitamin D receptor polymorphisms from three different loci were genotyped; *rs731236*, *rs1544410*, and *rs11568820*. Multivariate logistic regression models were used to determine odds ratios (OR) and 95% confidence intervals (CI) comparing aggressive to non-aggressive prostate cancer. Vitamin D deficiency (<20 ng/mL) significantly increased risk of aggressive disease (OR: 3.1, 95% CI: 1.03–9.57, *p*-value = 0.04). Stratification by total calcium showed high calcium levels (≥800 mg/day) modified this association (OR: 7.3, 95% CI: 2.15–47.68, *p*-interaction = 0.03). Genetic variant *rs11568820* appeared to increase the magnitude of association between deficient serum vitamin D and aggressive prostate cancer (OR: 3.64, 95% CI: 1.12–11.75, *p*-value = 0.05). These findings suggest that high incidence of aggressive prostate cancer risk in African American men may be due in-part to deficient levels of serum vitamin D. Other factors, including genetics, should be considered for future studies.

## 1. Introduction

Amongst men in the United States, prostate cancer is the most common malignancy. While incidence rates of prostate cancer have decreased over the years [1], studies have shown African American (AA) men to develop prostate cancer at a rate 1.5–1.9 times higher than their European American (EA) counterpart [2,3,4]. These racial differences are further emphasized by the increased diagnosis of aggressive prostate cancer [4,5]. Demographic characteristics, such as family history, socioeconomic status, access to medical care, other comorbidities, and diet and lifestyle have been shown to contribute to the increased burden of prostate cancer in AA men [2,6,7,8]. Recently, however, studies have focused on differences in serum 25-hydroxyvitamin D (25(OH)D) concentrations as a source of the disparate trends seen in this disease.

Critical to overall health, 25(OH)D plays a role in bone mineralization, diabetes mellitus, and multiple sclerosis [9,10]. The main source of 25(OH)D is derived from sunlight ultraviolet (UV)-B rays, accounting for over 90% of circulating levels [11,12,13]. High melanin, commonly seen in ethnic groups with dark skin, such as AA men, reduces the amount of UVB radiation absorbed in the skin, thus decreasing the concentration of 25(OH)D and increasing susceptibility to developing vitamin D deficiencies [14,15,16]. In the Health, Aging and Body Composition Study, comparison between AAs and EAs showed only 16% of older AA participants had serum 25(OH)D levels over 30 ng/mL, compared to 44% in EAs [17]. Data from the Prostate Cancer Prevention Trial determined AA men with higher vitamin D levels see a reduced risk in high-grade disease [18], while results in Afro-Caribbean men residing in the Caribbean indicate vitamin D insufficiency may contribute to increased prostate cancer risk [19,20]. Moreover, molecular studies suggest deficiencies in vitamin D overtime may lead to progression from pre-clinical to clinically aggressive forms of prostate cancer [21].

In addition to 25(OH)D, calcium intake is associated with risk of prostate cancer [22,23], where high levels are associated with an increase in metastatic disease [24]. In its association with vitamin D, results indicate that genes responsible for calcium absorption are regulated via the vitamin D receptor (*VDR*) [25,26]. In prostate cells that contain VDRs, response to the active form of vitamin D, 1-25 dihydroxyvitamin D (1,25(OH)_2_D) increases differentiation and decreases proliferation [27]. Conversely, high calcium has been shown to promote the proliferation of prostate cancer cells through calcium sensing receptors [28]. Although studies have examined associations of calcium and 25(OH)D on prostate cancer separately, few studies have accounted for the effects of both in a population of AA men.

Given the emergence of 25(OH)D and calcium as modifiable risk factors in prostate cancer development in AA men, and the paucity of studies in this racial population, we utilized a case-only study to explore associations between serum 25(OH)D, calcium, *VDR* genetic variants, and aggressive prostate cancer in AA men.

## 2. Materials and Methods

### 2.1. Participants

Unrelated men (*n* = 268) self-described as AA, age 40–85, were recruited between the years 2001 and 2004 from the Division of Urology at Howard University Hospital (HUH) in Washington, DC, USA. Incident prostate cancer cases (*n* = 155) were identified by a urologist within the division or the study coordinator, and confirmed by review of medical records. Clinical characteristics, including Gleason grade, prostate specific antigen (PSA), age at diagnosis, and family history, were obtained from medical records. Disease aggressiveness was defined as “non-aggressive” (Gleason grade < 7) or “aggressive” (Gleason grade ≥ 7); 58 men were categorized as non-aggressive, 46 were categorized as aggressive, and 51 did not have a Gleason score. These 58 men categorized as non-aggressive and 46 categorized as aggressive were examined in this study. The Howard University Institutional Review Board approved the study (approval code: IRB-02-MED-42), and written consent was obtained from all participants.

### 2.2. Serum 25-Hydroxyvitamin D Measurement and Nutritional Assessment

To measure serum 25(OH)D, fasting peripheral blood samples were collected at the time of recruitment. Serum samples were stored in small test tubes at −20 °C until 25(OH) D measurement. Total 25(OH) D was assessed by chemiluminescent immunoassay by the Associated Regional and University Pathologists laboratory in conjunction with the University of Utah. Serum 25(OH)D was defined as low if serum levels are below 20 ng/mL, or high if levels were ≥20 ng/mL.

Research coordinators conducted in-person interviews to obtain information on BMI, income, age, family history of prostate cancer, dietary intake, and alcohol and tobacco use from each participant. Dietary intake was assessed using the 1998 Block Food Frequency Questionnaire (FFQ), developed from an analysis of dietary recall data collected in the National Health and Nutrition Examination Survey (NHANES) [29]. The Block questionnaire has been validated for use in several populations including AAs [30]. Participants were asked about common serving size of food and beverages and frequency of consumption (never, 2–3 times per month, 1–2 times per week, 3–4 times per week, 5–6 times per week, or every day) in the past year. The completed FFQs were sent to NutritionQuest in Berkeley, CA, USA, where proprietary software was used to calculate estimates for total daily nutrient intakes for foods, beverages, and dietary supplements. From the FFQ, self-reported total calcium was assessed by combining dietary and supplemental calcium intake. Total calcium intake was defined as high if levels were ≥800 mg/day, and low if levels were <800 mg/day, based upon Institute of Medicine (IOM) average requirement.

### 2.3. Genotyping

Common polymorphisms on the vitamin D receptor, *rs11568820 (Cdx2)*, *rs1544410 (Bsm1)*, and *rs731236 (Taq1)*, were genotyped using the Sequenom MassARRAY iPLEX platform (Sequenom, San Diego, CA, USA), as previously described [31]. All three variants had a minor allele frequency of >10%, a genotyping rate of >95% and a Hardy-Weinberg *p*-value of >0.01 among all samples. Moreover, each variant was chosen for its potential interaction with vitamin D and calcium in the prostate, influencing the association between serum 25(OH)D levels and prostate cancer. Samples with a low genotyping rate (<95%) were removed from the analysis.

### 2.4. Statistical Analysis

Multivariate logistic regression models were used in this case-only study to estimate odds ratios (OR) and 95% confidence intervals (CI) for prostate cancer risk associations with serum 25(OH)D, comparing aggressive prostate cancer cases (*n* = 46) to non-aggressive cases (*n* = 58). Models were controlled for age (in years), body mass index (BMI) (continuous), smoking (yes/no), season of blood draw (UV high May-October vs. UV low November-April), and total calcium intake (continuous), as each of these variables resulted in material differences in odds ratios, by at least 10%. To examine the effect of modification by total calcium intake, we stratified our analysis by high total calcium (≥800 mg) and low total calcium (<800 mg). Stratified models were controlled for age, BMI, and smoking. A Wald test was used to determine the interaction term. To examine the effects of vitamin D receptor variants *rs1544410*, *rs731236*, or *rs11568820*, we added each variant as a covariate in our regression model.

All statistical tests were two-sided, and *p*-values less than 0.05 were considered to be statistically significant.

## 3. Results

Among 155 cases of prostate cancer, 58 cases were diagnosed as non-aggressive disease and 46 cases diagnosed as aggressive. Men with aggressive disease had a higher PSA and were more likely to have been former smokers (Table 1). Based upon cancer aggressiveness, a large percentage of both aggressive and non-aggressive cases had mean levels of serum 25(OH)D below deficient levels, as defined by the Institute of Medicine (IOM) (<20 ng/mL) [13]. There were no differences between aggressive and non-aggressive cases in participants’ age or BMI.

Risk of prostate cancer was assessed in association with deficient levels of 25(OH)D. Our results demonstrated that men diagnosed with aggressive prostate cancer and deficient in 25(OH)D, had a significant increase in risk compared to non-aggressive cases deficient in 25(OH)D (OR: 3.1, 95% CI: 1.03–9.57, *p*-value: 0.04) (Table 2A). Stratification by high and low levels of total calcium showed modification of the association between serum 25(OH)D deficiency and aggressive prostate cancer (Table 2B). Amongst cases with high total calcium, deficient levels of 25(OH)D showed strong associations with aggressive prostate cancer (OR: 7.3, 95% CI: 2.15–47.68). The significant interaction term (*p*-interaction = 0.03) demonstrates that high total calcium intakes modify the association between deficient levels of 25(OH)D and aggressive prostate cancer.

Examining the *VDR* SNPs, adding *rs1544410* and *rs731236* to the model did not change the magnitude of the association between aggressive prostate cancer and deficient levels of 25(OH)D (OR: 2.98, 95% CI: 0.98–9.05. *p*-value = 0.07; OR: 3.13, 95% CI: 0.99–9.60, *p*-value = 0.07, respectively) (Table 3). However, adding rs11568820 to the model increased the magnitude of the association between deficient 25(OH)D and aggressive prostate cancer (OR: 3.64, 95% CI: 1.12–11.75, *p*-value = 0.05).

## 4. Discussion

In this case-only study of 58 AA men with non-aggressive prostate cancer and 46 AA men with aggressive prostate cancer, we investigated associations of serum 25(OH)D and total calcium intake with aggressive prostate cancer risk. Our data indicated deficient levels of 25(OH)D significantly increased the risk of aggressive prostate cancer, compared to non-aggressive cases. We did not find an association between calcium and aggressive prostate cancer, however total calcium levels above 800 mg modified the association between deficient 25(OH)D and aggressive disease, where we reported a significant interaction.

While laboratory and epidemiological studies have shown evidence that supports decreased risk of prostate cancer with higher levels of serum 25(OH)D, recent studies have shown contradictory evidence. In the Prostate Cancer Prevention Trial, logistic regression models estimated increased overall prostate cancer risk with higher levels of serum 25(OH)D (OR: 1.18, 95% CI: 0.91–1.53, *p*-value = 0.08) [18]. Amongst aggressive disease with Gleason score 8–10, the positive association disappeared (OR: 0.50, 95% CI: 0.20–1.22, *p*-value = 0.22) [18]. Results from the Alpha-Tocopheral Beta-Carotene study, a case-control study of Finnish men, showed positive associations between increased serum 25(OH)D and overall prostate cancer (OR_Q5vs.Q1_: 1.36, *p*-trend = 0.03) [32]. Associations remained similar when stratified by aggressiveness (OR aggressive _Q5vs.Q1_: 1.70, *p*-trend = 0.02) [32]. In a study done in the Health Professionals Follow-up Study, 25(OH)D deficiency showed inverse associations with prostate cancer risk (OR: 0.62, 95% CI: 0.43–0.91) [33]. These studies, however, were conducted in populations that consisted of majority EA men. Amongst AA men, studies show multiple variables, including AA race and lack of vitamin D supplementation, increase the risk of 25(OH)D deficiency [9], subsequently increasing the risk of prostate cancer [34]. Interestingly, a recent study done in Jamaican men of African ancestry reported a positive association between high levels of 25(OH)D and total prostate cancer risk (OR_Q3vs.Q1_: 2.47, 95% CI: 1.20–4.90, *p*-value = 0.01) [35]. Analysis of FFQ results demonstrate Jamaican men report higher levels of serum 25(OH)D, averaging ≥ 30 ng/mL, compared to mean levels among AA men in the United States. The population differences seen between American AA men and Jamaican men could be explained by the U-shaped associations between aggressive prostate cancer risk and serum 25(OH)D. Serum concentrations below 20 ng/mL, as well as above 30 ng/mL, in AA men may have similar effects on disease risk. Further studies must be conducted to examine these trends.

Calcium intake has also played a significant role in prostate cancer development. Results from the California Collaborative Prostate Cancer Study show a 54% decrease in risk of aggressive disease amongst AA men with low levels of calcium intake [36]; EA men showed a 35% decrease in risk. A study in the San Francisco Bay and LA County areas showed positive associations between high dietary, total, and supplemental calcium intake and prostate cancer risk, in both total and aggressive disease [37]. The authors postulated the association between prostate cancer risk and high calcium may be an effect of *rs11568820* activity [37]. Studies have recognized the importance of the vitamin D receptor in disease development. As calcium mediates the effects of vitamin D in the body, variants of the VDR transcription factor binding sites have been shown to play an important role in the expression of genes that regulate prostate cancer development [38]. *VDR* variants, *Bsm1*, *Taq1*, *Apa1*, and *Fok1*, have been examined closely for their effects on prostate cancer risk [36]. Similar to other studies [39], we did not find associations between *rs1544410*, *rs731236*, and prostate cancer risk [39,40]. It is *rs11568820*, however, which has shown interesting effects.

Our study found high total calcium intake modified the association between deficient 25(OH)D levels and aggressive prostate cancer, increasing the risk of disease. Results from the Alpha-Tocopherol, Beta-Carotene (ATBC) Study contrast our results. Here, associations between high levels of serum 25(OH)D and total prostate cancer were stronger among men with higher intakes of total calcium (OR:_Q5vsQ1_ low calcium: 1.15, 95% CI: 0.75–1.75; OR_Q5vsQ1_ high calcium: 1.82, 95% CI: 1.20–2.76, *p*-interaction = 0.06) [32]. Moreover, our results are also in contrast with the report from Steck et al., which showed an inverse association between the highest tertile of serum 25(OH)D and aggressive prostate cancer amongst men with high levels of calcium [41]. As laboratory studies have shown that calcium promotes growth of prostate cancer cells [42], the results of our study are consistent with these findings. The differences we see with results from the ATBC Study could be due to race, but this does not explain the differences we see with Steck et al., as that study was stratified by race. A possible explanation for the modification by high total calcium may be related to genetic variation of the *VDR* that plays a role in calcium absorption. Located in the 5′ regulatory region of the *VDR* gene [43] *rs11568820* has been postulated to affect the *CDX2* transcription factor affinity for the *VDR* promoter [43]. These effects may induce changes to *VDR* expression in prostate cells and, thus, development of aggressive prostate cancer. *rs11568820* increased the risk of aggressive prostate cancer amongst 25(OH)D deficient levels, consistent with findings presented by Rowland et al. [37]. Here, the dominant A allele of *rs11568820* was associated with an increased risk of aggressive prostate cancer [37]. Studies, such as the Health Professionals Follow-up Study, show results in total prostate cancer only, where men with the *rs11568820* variant A allele who are deficient in 25(OH)D have a significant reduction in total prostate cancer risk (OR: 0.41, 95% CI: 0.21–0.82) [33]. Similar trends were seen among men with aggressive prostate cancer (OR: 0.18, 95% CI: 0.05–0.63) [33]. These differences in trends could be due to racial disparities, as our study was conducted in a population of all AA men, while the Health Professionals Follow-up Study consists of majority EA men. Moreover, these studies focus more on total prostate cancer, which is different from our unique focus on aggressive disease.

Interestingly, differences in calcium intake between AA and EA men also encompass differences in the *rs11568820* A allele expression. It is known that the *Cdx2 A* allele is more strongly associated with African ancestry [37]. Upon binding to the A allele on the *VDR*, the *rs11568820* transcription factor induces the expression of genes associated with calcium absorption, including calbindin. Increased calcium binds to calcium-sensing receptors on prostate cells, regulating proliferation and differentiation and, subsequently, the development of prostate cancer [44,45]. Given this pathway, further studies are needed to clarify the role increased calcium absorption plays within different races.

Our results showed similar effect sizes between two of our analyses: aggressive prostate cancer and 25(OH)D deficiency when *rs11568820* was added to the regression model, and aggressive disease and 25(OH)D deficiency without *rs11568820* in the model. Together, this data indicates a need for additional investigations into the association between aggressive prostate cancer and *VDR* SNPs, such as *rs11568820*.

Several limitations of our study should be noted. As a case-only study, our sample size of men was very small, with only 46 men with aggressive disease and 58 men with non-aggressive disease. Given the small number of cases being assessed, the results from our study must be cautiously interpreted, as we acknowledge this study is underpowered. Although other studies have assessed these outcomes in similar cohorts of similar sizes, this speaks to the need for studies that oversample minority and underrepresented participants, to increase the power to detect differences if they in fact exist. Our measurements of serum 25(OH)D and total calcium intake relied upon a single time point, which may have created an inadequate reflection of true levels. Another possible limitation is the use of the Block FFQ, which limits our accuracy of assessing dietary intake and may have introduced recall bias in the reporting. However, the Block FFQ has been validated for use in our study, and has been used in multiple epidemiologic studies assessing diet and disease associations. Strengths of our study include the oversampling of AA men with a focus on aggressive prostate cancer. Additionally, few epidemiologic studies have examined associations between serum 25(OH)D, calcium intake, and aggressive prostate cancer risk in a population of AA men.

## 5. Conclusions

In conclusion, our case-study produced data that supports a positive association between deficient serum 25(OH)D and aggressive prostate cancer. Moreover, this association is modified by high levels of total calcium. Although calcium intake alone does not have an association with aggressive disease, the *VDR* SNP rs11568820 increased the magnitude of the association between 25(OH)D and aggressive prostate cancer. However, serum 25(OH)D deficiency appears to play a larger role than calcium intake in aggressive prostate cancer. Future studies must explore modifiers of serum 25(OH)D and calcium in populations that recruit large numbers of AA participants. Additional investigations including genetic and ancestral differences in serum vitamin D and calcium concentrations should be done that include examinations of racial differences in additional genes involved in vitamin D metabolism, and calcium absorption.

## Figures and Tables

**Table 1:** Select characteristics by prostate cancer aggressiveness.

	Aggressive Cases	Non-Aggressive Cases	
Characteristics ^a^	(*n* = 46)	(*n* = 58)	*p*-value ^a^
Age, mean (SD)	66.4 (8.9)	64.2 (8.9)	0.21
BMI, kg/m^2^, mean (SD)	27.3 (5.8)	28.1 (4.7)	0.48
PSA, ng/mL, mean (SD)	66.5 (142.3)	21.1 (52.6)	0.05
Total calcium, mg, mean (SD)	999.2 (534.6)	809.3 (501.8)	0.08
Family history of prostate cancer, *n* (%) *			0.36
Yes	7 (15.2)	13 (22.4)	
No	29 (59.2)	33 (56.9)	
Income, *n* (%) *			0.35
<$30,000/year	26 (56.5)	26 (44.8)	
$30,001–$60,000/year	8 (17.4)	14 (24.1)	
≥$60,001/year	6 (13.4)	12 (20.7)	
Smoking status, *n* (%) *			0.01
No	9 (19.6)	24 (41.4)	
Yes, but quit	23 (50.0)	14 (24.1)	
Yes	10 (21.7)	11 (18.9)	
Serum 25(OH)D, ng/mL, *n* (%)			0.06
<20 ng/mL	32 (69.6)	30 (51.7)	
≥20 ng/mL	13 (28.3)	28 (48.3)	
Education *			0.55
High school	14 (30.4)	20 (35.5)	
Some college	2 (4.3)	6 (10.3)	
>4 years of college	3 (6.5)	8 (13.8)	
Ultraviolet (UV)-B Radiation exposure *			0.09
Low	9 (19.6)	20 (34.5)	
Medium and High	32 (69.6)	32 (55.2)	

^a^ *p*-values were calculated from independent sample *T* test for continuous variables and *χ*^2^ test for categorical variables; * Not all variable columns add up to 100% due to missing values.

**Table:** (**A**)

	*n* (Aggressive/non-Aggressive)	Age-Adjusted Odds Ratio	Age-Adjusted 95% CI	Fully Adjusted Odds Ratio ^a^	Fully Adjusted 95% CI	Fully Adjusted *p*-Value
Serum 25(OH)D						
≥20 ng/mL	13/25	1.0	reference	1.0	reference	
<20 ng/mL	31/27	2.3	0.92–5.22	3.1	1.03–9.57	0.04

**Table:** (**B**)

		*n* (Aggressive/Non-Aggressive)	Age-Adjusted Odds Ratio	Age-Adjusted 95% CI	Fully Adjusted Odds Ratio ^b^	Fully Adjusted 95% CI	Fully Adjusted *p*-Value
Low total calcium (<800 mg)							
	Serum 25(OH)D						
	≥20 ng/mL	9/13	1.0	reference	1.0	reference	
	<20 ng/mL	13/19	0.97	0.34–2.98	0.77	0.21–4.01	0.73
							
High total calcium (≥800 mg)							
	Serum 25(OH)D						
	≥20 ng/mL	4/12	1.0	reference	1.0	reference	
	<20 ng/mL	18/8	7.1	1.72–28.42	7.3	2.15–47.68	0.03

^a^ models adjusted for: age, BMI, smoking, total calcium, and season of blood draw; ^b^ models adjusted for: age, BMI, smoking, and season of blood draw.

**Table 3:** Multivariable odds ratios for the association between serum 25(OH)D and aggressive prostate cancer risk: individual models with each vitamin D receptor single- nucleotide polymorphisms (SNPs) included.

***rs731236***						
	***n* (Aggressive/Non-Aggressive)**	**Age-Adjusted Odds Ratio**	**Age-adjusted 95% CI**	**Fully Adjusted Odds Ratio ^a^**	**Fully Adjusted 95% CI**	***p*-Value**
Serum 25(OH)D						
≥20 ng/mL	13/25	1.0	reference	1.0	reference	
<20 ng/mL	30/27	2.19	0.98–5.13	2.98	0.98–9.05	0.07
						
***rs1544410***						
	***n* (Aggressive/Non-Aggressive)**	**Age-Adjusted Odds Ratio**	**Age-Adjusted 95% CI**	**Fully Adjusted Odds Ratio ^a^**	**Fully Adjusted 95% CI**	***p*-Value**
Serum 25(OH)D						
≥20 ng/mL	13/24	1.0	reference	1.0	reference	
<20 ng/mL	29/25	2.19	0.98–5.13	3.03	0.99–9.60	0.07
						
***rs11568820***						
	***n* (Aggressive/Non-Aggressive)**	**Age-Adjusted Odds Ratio**	**Age-Adjusted 95% CI**	**Fully Adjusted Odds Ratio ^a^**	**Fully Adjusted 95% CI**	***p*-Value**
Serum 25(OH)D						
≥20 ng/mL	13/25	1.0	reference	1.0	reference	
<20 ng/mL	28/23	2.19	0.98–5.13	3.64	1.12–11.75	0.05
						

^a^ models adjusted for: age, BMI, smoking, total calcium, *VDR* genetic variant, and season of blood draw.
